# P40 Dry eyes and a dry mouth: is it Sjögren’s syndrome?

**DOI:** 10.1093/rap/rkac067.040

**Published:** 2022-09-28

**Authors:** Ethan S Sen, Sharmila Jandial

**Affiliations:** Department of Paediatric Rheumatology, Great North Children's Hospital, Newcastle upon Tyne, United Kingdom; Faculty of Medical Sciences, Newcastle University, Newcastle upon Tyne, United Kingdom; Department of Paediatric Rheumatology, Great North Children's Hospital, Newcastle upon Tyne, United Kingdom; Faculty of Medical Sciences, Newcastle University, Newcastle upon Tyne, United Kingdom

## Abstract

**Introduction/Background:**

Sjögren's syndrome (SS) is a rare, multi-system, autoimmune disease characterised by immune-mediated inflammation and damage to salivary and lacrimal glands causing ‘sicca symptoms’ (dry eyes and mouth). There is a female predominance, and the median age of disease onset is 10 years, although the diagnosis is often delayed. Sicca symptoms are unusual in children presenting to primary or secondary care, and their presence may lead to referral to Paediatric Rheumatology with concerns about SS. Here we report two cases of children with dry eyes and mouth but without other features typical of SS.

**Description/Method:**

Case 1: A 16-month-old boy was referred to Paediatric Rheumatology with a history of dry eyes from infancy and a dry mouth. Previous Ophthalmology assessment showed blocked tear ducts. At age 14 months, dental review showed an unusually dry mouth. He was otherwise well with no history of rashes, joint pain or swelling. Examination was normal apart from mild eczema and particularly no obvious salivary gland enlargement. Initial blood tests showed a mild microcytic anaemia and raised erythrocyte sedimentation rate (ESR) at 24. Anti-nuclear antibodies (ANA), rheumatoid factor (RF) and antibodies against expressed nuclear antigens (ENA) were all negative. Salivary gland ultrasound was challenging but was reported as showing heterogenous parenchyma with numerous small hypoechoic/anechoic areas, suggestive of SS. Biopsy of this area did not show salivary gland tissue. A subsequent MRI showed absent parotid, submandibular and lacrimal glands.

Case 2: A 12-year-old boy was referred to Paediatric Rheumatology after previously presenting to General Paediatrics at the age of 8 years with a history of a dry mouth, dry eyes and thick nasal secretions. He suffered with angular cheilitis, mouth ulcers, phimosis and recurrent balanitis. Due to a history of dysphagia, he had previously been referred to Paediatric Gastroenterology who had diagnosed eosinophilic oesophagitis based on endoscopy findings and histology. On Rheumatology assessment, there was no history of rashes or joint symptoms, and examination revealed normal joints and no enlargement of his parotid glands. Other than a microcytic anaemia, blood tests were normal including ESR, negative ANA, RF and ENA. Ultrasound showed absent parotid and submandibular glands bilaterally.

**Discussion/Results:**

Both these boys presented with a history of dry eyes and a dry mouth but without other features of an inflammatory or autoimmune disease, and with negative autoantibodies. In Case 1, the salivary gland ultrasound was unexpectedly reported as showing some features of SS. Further investigation was felt to be necessary in light of his age, sex and unusual presentation, and was essential in making his diagnosis. On the basis of the MRI scan, he was diagnosed with congenital absence of salivary and lacrimal glands. This condition has been associated with variants in the fibroblast-growth factor 10 (*FGF10*) gene, and he was referred to Clinical Genetics for further evaluation. Analysis showed a novel variant, but it was unclear if this was pathogenic. Further genetic testing of his parents is awaited.

In Case 2, the ultrasound findings rapidly led to the diagnosis of congenital absence of salivary and lacrimal glands. He is awaiting an MRI scan to confirm the ultrasound findings. It is interesting that he presented with thick secretions, as opposed to absent secretions, and this may potentially explain his swallowing difficulties. Other conditions that lead to thick secretions such as cystic fibrosis and ciliary dyskinesia were considered but thought to be unlikely in light of his lack of respiratory symptoms.

These cases illustrate some of the challenges in the diagnostic process for rare diseases. The very early age of onset in Case 1 argued against SS and for a congenital disease, however the ultrasound imaging findings prompted further investigation. In Case 2, although the symptom onset was at an older age, the ultrasound findings of absent salivary glands pointed to the diagnosis of congenital absence of these glands.

**Key learning points/Conclusion:**

There are no validated diagnostic criteria for childhood SS. The American College of Rheumatology (ACR) and European League Against Rheumatism (EULAR) published classification criteria for SS in 2016 which include the following features: histopathologic evidence of focal lymphocytic sialadenitis on minor salivary gland biopsy; positive anti-SS-A/Ro antibodies; evidence of glandular dysfunction with decreased tear or saliva production. Referrals to Paediatric Rheumatology of a child with dry eyes and a dry mouth are relatively rare, but the priority is to diagnose or exclude SS.

An important differential diagnosis of SS is congenital absence of salivary and lacrimal glands. Features pointing to this diagnosis include a young age at symptom onset, absence of parotid gland swelling, absence of systemic symptoms and signs of autoimmune disease, and negative autoantibodies. Imaging with ultrasound or MRI confirms the diagnosis.

Some cases of congenital absence of salivary and lacrimal glands have been found to be associated with pathogenic variants in the *FGF10* gene. In this situation, the disease is known as aplasia of the lacrimal and salivary glands (ALSG; OMIM #180920). It is inherited in an autosomal dominant pattern with variable expressivity. If ALSG is suspected, referral to Clinical Genetics is recommended for testing and family counselling.

Management of patients with congenital absence of salivary and lacrimal glands is supportive and includes lubricating eye drops and attention to dental hygiene, with follow-up by an optician/ophthalmologist and dentist. Early recognition of the condition by Paediatric Rheumatologists can help to avoid excessive investigation and unnecessary immunosuppression.

